# Supplementary material for: Geographical variations of food insecurity and its associated factors in Bangladesh: Evidence from pooled data of seven cross-sectional surveys
Source: PLoS One. 2023 Jan 6;18(1):e0280157. doi: 10.1371/journal.pone.0280157 (PMC9821426; doi:10.1371/journal.pone.0280157)
Supplement: S1 Fig — (DOCX) [file pone.0280157.s001.docx]

**Figure S1**: Year-wise prevalence of food insecurity status of Bangladesh
