# Supplementary material for: Geographical variations of food insecurity and its associated factors in Bangladesh: Evidence from pooled data of seven cross-sectional surveys
Source: PLoS One. 2023 Jan 6;18(1):e0280157. doi: 10.1371/journal.pone.0280157 (PMC9821426; doi:10.1371/journal.pone.0280157)
Supplement: S1 Table — (DOCX) [file pone.0280157.s002.docx]

**Table S1:**  Household hunger scale questionnaire

| **Household Hunger Scale (Food Security)** | |
| --- | --- |
| Ques. No. | **Questions** |
| Q.1 | In the past 4 weeks (30 days), how often did you worry that your household would not have enough food days? |
| Q.2 | In the past 4 weeks (30 days), how often were you or any household member not able to eat the kinds of foods that you or s/he preferred because of a lack of resources? |
| Q.3 | In the last 4 weeks (30 days), how often did you or any household member have to eat a limited variety of foods due to a lack of resources? |
| Q.4 | In the last 4 weeks (30 days), how often did you or any household member have to eat some foods that you or s/he really did not want to eat because of a lack of resources to obtain other types of food? |
| Q.5 | In the last 4 weeks (30 days), how often did you or any household member have to eat a smaller meal than you or s/he felt was needed because there was not enough food? |
| Q.6 | In the last 4 weeks (30 days), how often did you or any other household member have to eat fewer meals in a day because there was not enough food? |
| Q.7 | In the last 4 weeks (30 days), how often was there ever no food to eat of any kind in your household because of lack of resources to get food? |
| Q.8 | In the last 4 weeks (30 days), how often did you or any household member go to sleep at night hungry because there was not enough food? |
| Q.9 | In the last 4 weeks, how often did you or any household member go a whole day and night without eating anything because there was not enough food? |
