# Supplementary material for: Geographical variations of food insecurity and its associated factors in Bangladesh: Evidence from pooled data of seven cross-sectional surveys
Source: PLoS One. 2023 Jan 6;18(1):e0280157. doi: 10.1371/journal.pone.0280157 (PMC9821426; doi:10.1371/journal.pone.0280157)
Supplement: Attachment — Submitted filename: Response to Reviewers.docx [file pone.0280157.s006.docx]

**Summary and general evaluation**

While the authors have addressed some of the comments, I note below where the authors still need to strengthen their arguments and incorporate additional changes to the manuscript.

**Main comments**

1) **Contribution**: While the authors have made some attempts to contrast their findings to that of Hossain et al. (2020), the paper’s contribution is not fully fleshed out yet and needs to be clearer and more explicit (beyond stating the use of more recent data). The writing needs to be re-framed so that, as a reader, it is clear what additional thing we learn from this paper relative to existing studies (whether its methodological innovation or otherwise). Other studies find similar results that food insecurity is prevalent in northern and southern parts of the country and that education and poverty are significant correlates of food insecurity, so the value addition needs to be clearer.

***Response:*** *Thank you for noting these. This study was conducted exclusively in the BRAC’s MIYCN programme areas and this study intended to understand the food insecurity in the areas despite having programmatic efforts in improving health outcomes. In addition, our study used Household Food Insecurity Access Scale whereas Hossain et. al. (2020) used per calorie intake method. We emphasized MIYCN programme areas in the revised version which we think will distinguish our study from others.*

*We also reframed our manuscript, particularly in the discussion section for a clear understanding of the readers.*

2) **Method and measurement**: Please write down the main estimating equation in the methods section. While the control variables have been described much better now, the equation helps the reader to understand more clearly what you are capturing in your analysis.

It’s good that there are additional details on the construction of the main outcome variable. However, lines 169 and 170 seem to provide conflicting numbers on whether the response to each question was scored from 0 to 30 or 0 to 27.

***Response:*** *Thank you for your valuable comments. In the revised manuscript, we have added the equation as follows—*

*The general equation of the multivariable logistic regression model is:*

$$y=log \left[ \frac{p}{1-p} \right]=\alpha_{0}+\beta_{1}x_{1}+ \beta_{1}x_{2}+\beta_{1}x_{2}+\ldots+\beta_{n}x_{n}$$

*Where:*

*y = Outcome variable (food insecurity status)*

*p = probability of household to be food insure*

$\alpha_{0}$*= Intercept (constant)*

$\beta_{1}$*,* $\beta_{2}$*,* $\beta_{3},$*……*$\beta_{n}$*= Coefficients of the respective independent variables*

$x_{1}, x_{2}$*,* $x_{3}, \ldots x_{n}$ *= Independent variables (Household size, household head’s age, education of household head…survey year, etc.)* ***(page 10, lines 196-204)***

*Also, we have revised the scoring of the outcome variable as follows—*

*The questions were asked on how often the level of concern, lack of access to, variety, and quantity of food happened. The response to each question was between 0 to 30. We made scoring based on the responses as 0=0, 1-2=1, 3-10=2, and 11-30=3. Thus, the score ranged from 0 to 27 for 9 questions. We then categorized the scores as 0-1=food secure, score 2/7=mild food insecure, 8/14=moderate food insecure, and score 15-27=severe food insecure.* ***(page 8, lines 162-166).***

3) **Exposition**: Thank you for addressing some of the select copy editing notes I made last time. However, there is still significant scope for improving the exposition of the paper; for instance, there are still copy edits that have not been addressed [As an example, text in lines 209–210, lines 383–386]. There are some repetitive sentences in the manuscript. While I note some examples, the entire paper can benefit from further copy-editing.

***Response:*** *Thank you for your valuable suggestion. In the revised manuscript, we made substantial edits. In addition, we removed the repetitive sentences.*

**Minor comments**

1) The tables have additional information now, which is helpful, but it is not yet fully self-contained. It should note what is being captured in the main outcome variable. A reader should be able to understand what is being estimated in the table based on the table notes.

***Response:*** *Thanks a lot. We have added information in the footnote of Table 1 and additional information about the outcome variable in the footnote of Table 2.*
