# Supplementary material for: Geographical variations of food insecurity and its associated factors in Bangladesh: Evidence from pooled data of seven cross-sectional surveys
Source: PLoS One. 2023 Jan 6;18(1):e0280157. doi: 10.1371/journal.pone.0280157 (PMC9821426; doi:10.1371/journal.pone.0280157)
Supplement: Attachment — Submitted filename: Reviewer comments.docx [file pone.0280157.s003.docx]

**Summary and general evaluation**

This paper aims to identify geographical variation in food insecurity at the district level and its correlates in Bangladesh. The authors utilize a GEE model to identify household-level factors associated with food security and hot spot analysis for spatial mapping of food insecurity. The main finding identifies the education of household heads or caregivers and poverty to be the key correlates of the food insecurity status of a household. While the results are interesting, the authors can better explain the contribution of this paper and clarify the details of the technical analysis, which I elaborate on below.

**Main comments**

1) This paper’s contribution could be made more explicit. Towards the end of the introduction, the authors refer to existing studies on food insecurity in Bangladesh. The literature discussion would be more effective if used to contrast this paper’s relative contribution better. Hossain et al. (2020) also map the prevalence, gap, and severity of food insecurity in Bangladesh at the district level using survey and census data to find northern and southern parts more vulnerable. It would be useful to compare and illustrate the value addition of the methods used and findings of this paper (the indicator of food security is also different). For instance, the hot spot analysis identifies the central region as vulnerable to food insecurity in this paper. Is this due to the method used, year-specific changes (Hossain et al. (2020) use data from 2010 and 2011 whereas this paper uses data from 2015­­–2018) or the choice of outcome variable? Even if the reasons for the central region findings are hard to pin down, the value addition of this paper should be strengthened in the introduction.

2) Measurement of food insecurity: There need to be more details on how the main outcome variable is constructed. It is not clear how the nine questions are used and what criterion is applied to determine the different levels of food insecurity. Are the hot spot analysis results sensitive to how the food insecurity criterion is applied? It would be useful also to discuss the advantages or limitations of what the outcome variable captures compared to alternate measures such as the per capita calorie intake used by the Bangladesh Bureau of Statistics (Hossain et al. 2020). If data is available, it would be interesting to see the robustness of results to alternative definitions of measuring food insecurity.

3) It would be helpful to specify the main estimating equation in the methods section to clarify the control variables, including district and survey fixed effects. There can be additional discussion on why GEE models are used in this context.

4) In the main GEE model, all covariates relevant in affecting food security (based on theory or previous literature) should be presented in the main model (regardless of statistical significance). Line 178 suggests that only statistically significant covariates from the simple GEE model were retained in the final multivariable model.

5) Is it possible to compare the magnitude of the estimates of food insecurity with other studies to contextualize the results? There is some reference to other estimates from the literature in lines 284­–286. However, additional details are required to have more context regarding the time period and the comparable statistics with this study (if any).

6) In Figure 4, what does the blue area indicate? The map legend does not specify it, so it is hard to interpret.

7) For the summary statistics in Table 1, it would be good to report the standard deviation of the variables.

8) The exposition of the paper can be improved with some copy editing. I cite some select references, for e.g., the text in lines 71–73, 186–187, 197–199, 292 needs to be revised.

**Minor comments**

1) The abstract states that the food insecurity hotspots are in Bangladesh’s northwestern and coastal regions. However, when describing areas for targeted interventions, it refers to northwestern, central, and coastal regions. The main analysis indicates central regions also to be vulnerable to food insecurity. The abstract can be updated accordingly.

2) In the abstract and line 113, it ought to be BRAC instead of BRAC’s.

3) In line 138, it is noted that the method adopted is similar to the approach of the EPI. The way it is phrased at present, it is unclear what this refers to.

4) Coastal spelled incorrectly in line 248.

5) Add detailed notes to the tables and figures to be self-contained, including information on the outcome variable.

6) Drop the open bracket in line 134 before PSUs.

**Reference:**

Hossain, M. J., Das, S., Chandra, H., & Islam, M. A. (2020). Disaggregate level estimates and spatial mapping of food insecurity in Bangladesh by linking survey and census data. *PloS One*, *15*(4), e0230906.
