# Supplementary material for: Geographical variations of food insecurity and its associated factors in Bangladesh: Evidence from pooled data of seven cross-sectional surveys
Source: PLoS One. 2023 Jan 6;18(1):e0280157. doi: 10.1371/journal.pone.0280157 (PMC9821426; doi:10.1371/journal.pone.0280157)
Supplement: Attachment — Submitted filename: Reviewer comments.docx [file pone.0280157.s005.docx]

**Summary and general evaluation**

While the authors have addressed some of the comments, I note below where the authors still need to strengthen their arguments and incorporate additional changes to the manuscript.

**Main comments**

1) **Contribution**: While the authors have made some attempts to contrast their findings to that of Hossain et al. (2020), the paper’s contribution is not fully fleshed out yet and needs to be clearer and more explicit (beyond stating the use of more recent data). The writing needs to be re-framed so that, as a reader, it is clear what additional thing we learn from this paper relative to existing studies (whether its methodological innovation or otherwise). Other studies find similar results that food insecurity is prevalent in northern and southern parts of the country and that education and poverty are significant correlates of food insecurity, so the value addition needs to be clearer.

2) **Method and measurement**: Please write down the main estimating equation in the methods section. While the control variables have been described much better now, the equation helps the reader to understand more clearly what you are capturing in your analysis.

It’s good that there are additional details on the construction of the main outcome variable. However, lines 169 and 170 seem to provide conflicting numbers on whether the response to each question was scored from 0 to 30 or 0 to 27.

3) **Exposition**: Thank you for addressing some of the select copy editing notes I made last time. However, there is still significant scope for improving the exposition of the paper; for instance, there are still copy edits that have not been addressed [As an example, text in lines 209–210, lines 383–386]. There are some repetitive sentences in the manuscript. While I note some examples, the entire paper can benefit from further copy-editing.

**Minor comments**

1) The tables have additional information now, which is helpful, but it is not yet fully self-contained. It should note what is being captured in the main outcome variable. A reader should be able to understand what is being estimated in the table based on the table notes.

**Reference:**

Hossain, M. J., Das, S., Chandra, H., & Islam, M. A. (2020). Disaggregate level estimates and spatial mapping of food insecurity in Bangladesh by linking survey and census data. *PloS One*, *15*(4), e0230906.
